# Systematic review of clinical practice guidelines on acupuncture for chronic musculoskeletal pain

**DOI:** 10.1186/s12906-025-05070-y

**Published:** 2025-09-01

**Authors:** Leonard Ho, Cyrus Ngo Tin Lai, Haiyong Chen, Sheung Wai Law, Edwin Chau Leung Yu, Fiona Pui Yan Lam, Yi Chung Cheung, Irene Xinyin Wu, Samuel Yeung Shan Wong, Regina Wing Shan Sit

**Affiliations:** 1 Jockey Club School of Public Health and Primary Care, Faculty of Medicine, The Chinese University of Hong Kong, Hong Kong Special Administrative Region, Shatin, People’s Republic of China; 2 School of Chinese Medicine, Faculty of Medicine, The University of Hong Kong, Hong Kong Special Administrative Region, Shatin, People’s Republic of China; 3 Department of Orthopaedics & Traumatology, Faculty of Medicine, The Chinese University of Hong Kong, Hong Kong Special Administrative Region, Shatin, People’s Republic of China; 4 Hong Kong Institute of Integrative Medicine, Faculty of Medicine, The Chinese University of Hong Kong, Hong Kong Special Administrative Region, Shatin, People’s Republic of China; 5 School of Chinese Medicine, Hong Kong Special Administrative Region, Hong Kong Baptist University, Shatin, People’s Republic of China; 6 Sin-Hua Herbalists’ and Herb Dealers’ Promotion Society Limited, Hong Kong Special Administrative Region, Shatin, People’s Republic of China; 7 Xiangya School of Public Health, Central South University, Changsha, People’s Republic of China; 8 School of Public Health Building, Prince of Wales Hospital, Shatin, New Territories, Hong Kong

**Keywords:** Practice guideline, Acupuncture, Complementary therapies, Musculoskeletal pain, Systematic review

## Abstract

**Introduction:**

Acupuncture is increasingly utilised in primary care to manage chronic musculoskeletal pain, supported by a growing body of evidence. This rising adoption has driven demand for clinical practice guidelines (CPGs). We summarised the characteristics of recent acupuncture CPGs for osteoarthritis, low back pain, neck pain, and shoulder pain, and critically appraised their methodological quality.

**Methods:**

We searched nine databases to identify acupuncture CPGs published from January 2014 to November 2024. Eligible CPGs were required to be developed by guideline committees and to include evidence-informed recommendations linked to clearly defined levels of evidence. Two independent reviewers extracted CPG characteristics and assessed methodological quality using the Appraisal of Guidelines for Research and Evaluation II (AGREE II) instrument.

**Results:**

Of the 1,999 records screened, 17 CPGs were included, encompassing 35 recommendations. Shoulder pain was the most addressed condition (*n* = 14), followed by low back pain (*n* = 11), osteoarthritis (*n* = 8), and neck pain (*n* = 2). Various types of acupuncture were considered, with manual acupuncture featuring in most (*n* = 26) recommendations. Overall, 60% of the recommendations supported the use of acupuncture, comprising 5.7% strong recommendations and 54.3% weak or conditional recommendations. In contrast, 22.9% of recommendations offered no explicit guidance, while 17.1% advised against its use. Methodological assessment classified 10 CPGs as high quality, while seven were of moderate quality.

**Conclusions:**

Contradictions exist among the included CPGs regarding whether acupuncture should be recommended for routine practice, potentially reflecting differences in clinical and cultural contexts. Local CPGs should be developed using rigorous methodology, ensuring the involvement of local stakeholders. An AGREE II extension should be developed for the methodological quality assessment of acupuncture CPGs.

**Supplementary Information:**

The online version contains supplementary material available at 10.1186/s12906-025-05070-y.

## Introduction

According to the Global Burden of Disease 2021, musculoskeletal disorders, represented by rheumatoid arthritis, osteoarthritis, low back and neck pain, and gout, accounted for approximately 162 million disability-adjusted life years, ranking first among all Level 2 causes for years of healthy life lost due to disability in both sexes combined [1]. Chronic pain resulting from musculoskeletal disorders adversely effects individuals’ physical, psychological, and social wellbeing, while also imposing a substantial financial burden on countries and healthcare systems worldwide [2]. In the United States alone, chronic pain, including musculoskeletal pain, is estimated to cost at least USD 560 billion per year in direct healthcare expenses and productivity losses [3]. Given its impact, it is increasingly recognised as one of the public health priorities, necessitating a more comprehensive understanding and targeted policy responses [4, 5]. 

Chronic musculoskeletal pain is typically managed at the primary care level with a range of pharmacological and non-pharmacological options. Pharmacological approaches generally include non-opioid analgesics (e.g., paracetamol, non-steroidal anti-inflammatory drugs, opioids, and adjuvant medications (e.g., antidepressants, muscle relaxants, corticosteroids) [6, 7]. Non-pharmacological interventions recommended in clinical practice encompass supervised exercise programmes, psychological therapies, electrical physical modalities (e.g., transcutaneous electrical nerve stimulation), and acupuncture [6, 7]. Education provided by primary care practitioners is also essential in empowering individuals with chronic musculoskeletal pain to effectively manage their condition, equipping them with relevant knowledge and skills to optimise clinical outcomes and quality of life [6, 7]. 

Acupuncture is practised and officially regulated in over 100 World Health Organization Member States, particularly in countries across Asia, as an intervention of traditional Chinese medicine (TCM) and some other practices of complementary and alternative medicine (CAM) [8]. Decades of research have been dedicated to evaluating acupuncture’s effectiveness and adverse events to support its application in clinical practice for various conditions and disorders [9, 10]. A recent individual patient data meta-analysis of high-quality trials indicated that acupuncture has a clinically relevant and persistent effect in the management of osteoarthritis, chronic headache, shoulder pain, and non-specific musculoskeletal pain, when compared to no acupuncture or sham acupuncture [11]. The increased uptake of acupuncture in clinical practice and the accumulation of supporting evidence have led to a growing demand for clinical practice guidelines (CPGs) [12], with the aims to provide evidence-based recommendations on indications, treatment duration, relevant acupoints, and information on potential adverse events and contraindications.

A 2018 bibliometric analysis examined trends in acupuncture use and the conditions addressed within its included 1,311 CPGs published between 1991 and 2017 [13]. Similarly, a recent systematic review evaluated 133 acupuncture CPGs published from 2010 to 2020 [14], with 39 focused on musculoskeletal and connective tissue disorders. However, neither of them critically appraised the level of evidence underpinning the recommendations in these CPGs. Instead, they provided either a narrative overview of publication characteristics [13, 14] or, at most, evaluated the CPGs methodological quality and addressed whether the CPG developers had conducted an assessment of evidence level in terms of “Yes” or “No” only [14]. This omission leaves an important gap, as it remains unclear whether the recommendations are sufficiently robust to inform clinical decision-making.

Therefore, it is important to critically appraise existing CPGs to understand how acupuncture is positioned in the field of chronic musculoskeletal pain conditions. Rigorous guideline appraisal can identify areas of consensus, highlight inconsistencies, and inform efforts to improve guideline development and implementation.

This systematic review aims to synthesise acupuncture CPGs for chronic musculoskeletal pain, focusing on their recommendations about pain relief, physical function improvement, and quality of life improvement. It also seeks to provide a standardised summary of the levels of evidence supporting these recommendations and their corresponding grades, along with a critical appraisal of the CPGs’ methodological quality.

## Methods

We conducted this systematic review based on PRISMA (Preferred Reporting Items for Systematic Reviews and Meta-Analyses) guidelines [15]. The review protocol was registered in PROSPERO (CRD42024620086).

### Eligibility criteria

We included CPGs focusing on the use of acupuncture for three of the most prevalent chronic musculoskeletal pain conditions—osteoarthritis, low back pain, and neck pain [1]—as well as shoulder pain, another frequently encountered musculoskeletal pain in primary care [16]. Regardless of the publication language, eligible CPGs should be developed by guideline development committees or equivalent bodies and to provide detailed information on systematic literature search and evidence-informed recommendations linked to clearly defined levels of evidence. Those recommendations should focus on clinical outcomes including pain alleviation, as well as improvements in physical function and quality of life. No restrictions were applied regarding the applicable populations, types of acupuncture (e.g., manual acupuncture, electro-acupuncture) or intervention comparators (e.g., placebo, anti-inflammatory agents). For updated versions of CPGs, only the most recent publications were included.

### Search strategy and selection criteria

We searched for eligible CPGs published between January 2014 and November 2024 across nine electronic databases, including Medline (Ovid), Embase (Ovid), Trip Medical Database, National Institute for Health and Care Excellence Database, Guidelines International Network, China National Knowledge Infrastructure, WanFang, SinoMed, and Database of Chinese Sci-Tech Periodicals. In addition, we performed a hand search of the reference lists of excluded conference abstracts. All records were imported into Covidence (https://www.covidence.org/) (Veritas Health Innovation, Melbourne, Australia), where title and abstract screening, as well as full-text assessment, were conducted by two reviewers (LH, CNTL) independently. Conflicts were resolved by consensus or, if necessary, by a third reviewer (RWSS).

### Data extraction

A reviewer extracted three levels of information from each CPG, which were subsequently validated by another reviewer. CPG-level information included the first author, publication year, publication type (report or journal article), publishing organisation, country of origin, funding source, CPG type (acupuncture-specific, CAM, or comprehensive), and the chronic musculoskeletal pain addressed. Intervention-level information comprised the type of acupuncture, selection of acupoints, duration of treatment, potential adverse events, applicable populations, and the healthcare settings in which the interventions should be delivered. Recommendation-level information involved clinical outcome(s) and effectiveness summaries, the assessment system adopted for formulating recommendations and rating quality of evidence, the reported strength of recommendation, the reported level of evidence, and comparator(s) of the recommendation.

### Evaluation of levels of evidence and grades of recommendations

The included CPGs employed various assessment systems to evaluate and report the quality of the evidence underpinning their recommendations and the rigour of the recommendations themselves. To address this variability, we adopted a standardised approach proposed by the Oxford Centre for Evidence-Based Medicine (CEBM) to assess the levels of evidence and grades of recommendations within the CPGs [17, 18]. 

Specifically, the level of evidence supporting a recommendation was rated based on the “Hierarchy of Evidence”, with systematic reviews of randomised controlled trials representing the highest level (i.e., Level 1) of evidence, while expert opinions without explicit critical appraisal are considered the lowest level (i.e., Level 5) [17, 18]. A minus sign (“-”) was used to indicate instances where an effect estimate had a wide confidence interval or a systematic review exhibited high heterogeneity, signifying a lack of conclusive evidence. In other words, a systematic review with high heterogeneity would be rated at Level 1-. The grade of a recommendation was determined by the level(s) of supporting evidence and the consistency of that evidence [17, 18]. Grade “A” recommendations are those supported by consistent Level 1 evidence, whereas Grade “D” recommendations are based on Level 5 evidence or evidence of any level that is troublingly inconsistent or inconclusive. The evaluation process was conducted independently by two reviewers (LH, CNTL), with any disagreements resolved through discussion.

### Assessment of guideline methodological quality

We used the Appraisal of Guidelines for Research and Evaluation II (AGREE II) instrument to assess the methodological quality of each included CPG [19]. The AGREE II instrument comprises 23 key items, organised within six domains, applicable to various disease areas and stages across the healthcare continuum. These six domains are: (i) scope and purpose; (ii) stakeholder involvement; (iii) rigour of development; (iv) clarity of presentation; (v) applicability; and (vi) editorial independence. Each key item was rated independently by two reviewers (LH, CNTL) on a 7-point Likert scale (1 = strongly disagree to 7 = strongly agree). Domain scores were calculated by summing the scores of the key items within each domain across two reviewers and then scaling this total as a percentage of the maximum possible score for that domain [19]. For example, if one reviewer strongly agreed with all three key items in the domain “scope and purpose” (7 points * 3 items = 21 points), while the other reviewer provided neutral ratings (4 points * 3 items = 12 points), the score for this domain would be calculated as 75% ((33 [total obtain score] – 6 [minimum possible score]/(42 [maximum possible score] – 6 [minimum possible score]) * 100%). Upon completing the 23 items, the reviewers were required to provide two overall assessments of each CPG in terms of its overall quality (on a 7-point Likert scale) and whether to recommend the use of the CPG (Yes/Yes, with modifications/No). The overall quality score was calculated using the same approach applied for domain score calculation.

As there are no established cut-off scores for the domains or overall quality, we classified domain and overall scores below 50.0% as indicative of low quality, scores between 50.0% and 80.0% as moderate quality, and scores above 80.0% as high quality [14]. 

### Data analysis

All extracted information and assessment results were appropriately tabulated and summarised narratively. No quantitative data synthesis was performed.

## Results

### Study selection

**Fig. 1:**
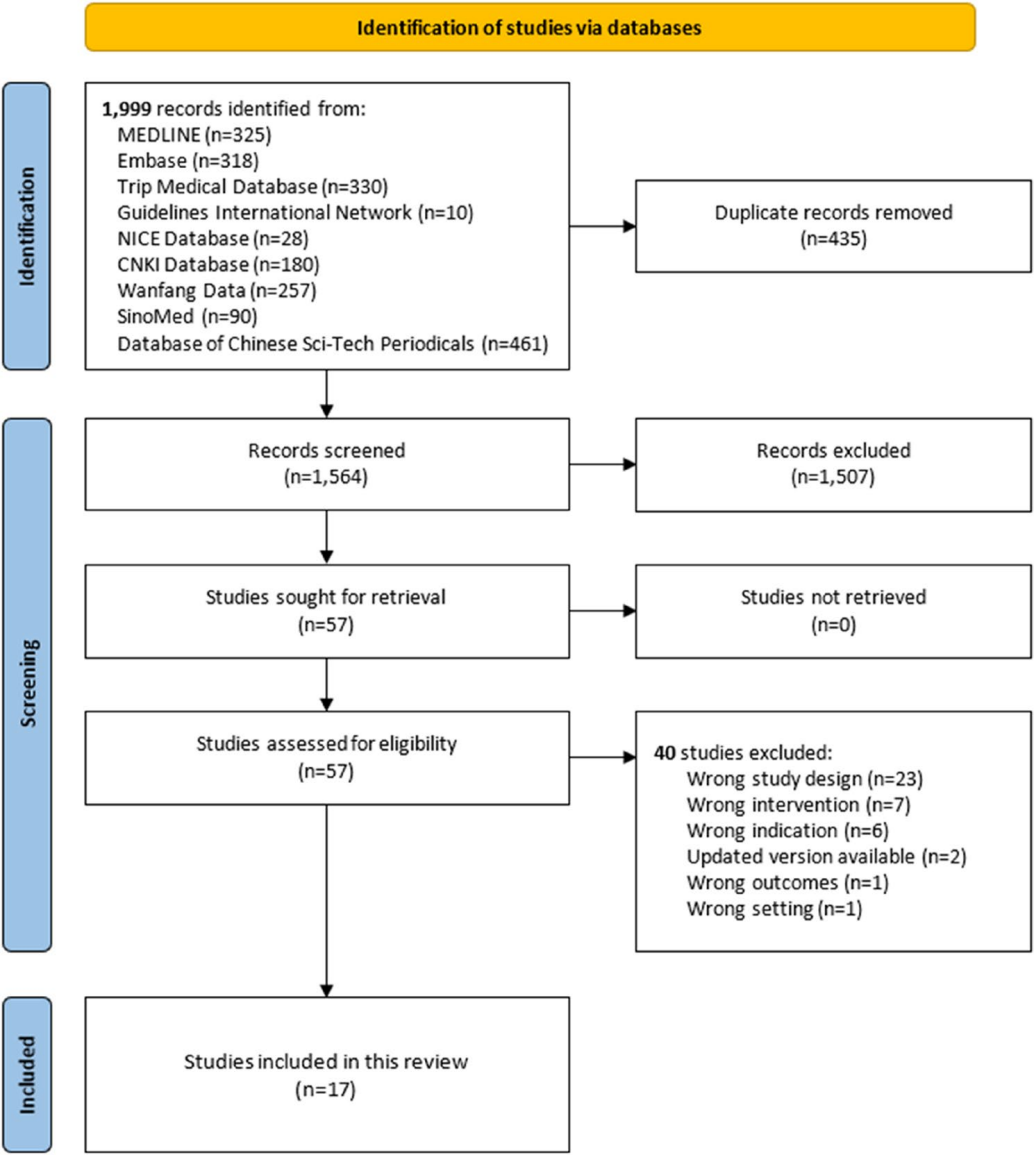
Flow of literature search and selection

The literature search identified 1,999 records. After deduplication, 1,564 records were screened by title and abstract, and 57 full-text documents were assessed. Finally, 17 eligible CPGs focusing on chronic musculoskeletal pain management involving acupuncture were included. Figure 1 illustrates the literature selection process.

### Characteristics of the included clinical practice guidelines

Table 1 summarises the characteristics of the included CPGs. These 17 CPGs were published between 2017 and 2023, with nine (52.9%) appearing in peer-reviewed journals. The United States contributed the highest number of CPGs (*n* = 6; 35.3%), followed by Canada, the People’s Republic of China, and the United Kingdom, each producing two. All of them were funded by professional organisations, governments, or government-funded projects. Most (*n* = 14; 82.4%) were comprehensive CPGs, addressing interventions from more than one medical practice (e.g., conventional medicine, TCM), while two (11.8%) focused exclusively on TCM and one (5.9%) on traditional Korean medicine. Eight (47.1%) CPGs focused on the management of chronic low back pain and conditions contributing to low back pain, such as lumbar spinal stenosis and lumbar radiculopathy. Six (35.3%) CPGs addressed the management of hip and/or knee osteoarthritis, or osteoarthritis in general. Two (11.8%) CPGs each focused on the management of chronic neck pain (including cervical radiculopathy) and shoulder pain (including frozen shoulder) with acupuncture.

**Table 1:** Characteristics of the 17 included clinical practice guidelines

First author	Title	Year	Type of publication	Organisation	Country of origin	Funding source	Type of CPG	Chronic musculoskeletal pain
1. Qin	Traditional Chinese medicine for frozen shoulder: An evidence-based guideline	2023	Journal article	Not published by particular organisations	People’s Republic of China	The National Traditional Chinese Medicine Inheritance and Innovation Team Project; The Chinese Medicine Evidence-based Capacity Improvement Project	CAM (Traditional Chinese medicine)	Frozen shoulder
2. The American Academy of Orthopaedic Surgeons	American Academy of Orthopaedic Surgeons management of osteoarthritis of the knee (non-arthroplasty) evidence-based clinical practice guideline	2021	Report	The American Academy of Orthopaedic Surgeons	United States	The American Academy of Orthopaedic Surgeons	Comprehensive	Knee osteoarthritis
3. Shirado	Formulation of Japanese Orthopaedic Association (JOA) clinical practice guideline for the management of low back pain - The revised 2019 edition	2022	Journal article	The Japanese Orthopaedic Association	Japan	The Japanese Orthopaedic Association; The Japanese Society of Lumbar Spine Disorders	Comprehensive	Low back pain
4. The North American Spine Society	Evidence-based clinical guidelines for multidisciplinary spine care: Diagnosis & treatment of low back pain	2020	Report	The North American Spine Society	United States	The North American Spine Society	Comprehensive	Low back pain
5. Bussières	Non-surgical interventions for lumbar spinal stenosis leading to neurogenic claudication: A clinical practice guideline	2021	Journal Article	The Canadian Chiropractic Guideline Initiative; The Bone and Joint Canada; The International Taskforce on Diagnosis and Management of Lumbar Spinal Stenosis	Canada	The Canadian Chiropractic Research Foundation	Comprehensive	Lumbar spinal stenosis
6. The Department of Veterans Affairs and Department of Defense Evidence-Based Practice Work Group	VA/DoD clinical practice guideline: Diagnosis and treatment of low back pain	2022	Report	VA and DoD Evidence-Based Practice Work Group	United States	The VA Evidence Based Practice, Office of Quality and Patient Safety	Comprehensive	Low back pain
7. Kjaer	National clinical guidelines for non-surgical treatment of patients with recent onset neck pain or cervical radiculopathy	2017	Journal Article	The Danish Health Authority	Denmark	The Danish Finance Act	Comprehensive	Cervical radiculopathy
8. Stochkendahl	National clinical guidelines for non-surgical treatment of patients with recent onset low back pain or lumbar radiculopathy	2017	Journal Article	The Danish Health Authority	Denmark	The Danish Finance Act	Comprehensive	Lumbar radiculopathy
9. Choi	Evidence Based (GRADE Approach) Korean Medicine clinical practice guidelines of manual acupuncture for the treatment of shoulder pain	2017	Journal Article	The Society of Korean Medicine Rehabilitation	South Korea	The Korea Institute of Oriental Medicine	CAM (Traditional Korean medicine)	Shoulder pain
10. The Department of Veterans Affairs and Department of Defense Evidence-Based Practice Work Group	VA/DoD clinical practice guideline for the non-surgical management of hip & knee osteoarthritis	2020	Report	VA and DoD Evidence-Based Practice Work Group	United States	The VA Evidence Based Practice, Office of Quality and Patient Safety	Comprehensive	Hip osteoarthritis; knee osteoarthritis
11. The National Institute for Health and Clinical Excellence	Low back pain and sciatica in over 16s: Assessment and management	2020	Report	The National Institute for Health and Clinical Excellence	United Kingdom	The UK Department of Health and Social Care	Comprehensive	Low back pain
12. Luo	Acupuncture for treatment of knee osteoarthritis: A clinical practice guideline	2023	Journal Article	Not published by particular organisations	People’s Republic of China	The National Science Fund for Distinguished Young Scholars; The Sichuan Provincial Central Government Guides Local Science and Technology Development Special Project; The Fundamental Research Funds for the Central Public Welfare Research Institutes; The Innovation Team and Talents Cultivation Program of National Administration of Traditional Chinese Medicine	CAM (Traditional Chinese medicine)	Knee osteoarthritis
13. Korownyk	PEER simplified chronic pain guideline: Management of chronic low back, osteoarthritic, and neuropathic pain in primary care	2022	Journal Article	The College of Family Physicians of Canada	Canada	The College of Family Physicians of Canada; The Ontario College of Family Physicians; The Alberta College of Family Physicians; The Saskatchewan College of Family Physicians	Comprehensive	Low back pain
14. Qaseem	Noninvasive treatments for acute, subacute, and chronic low back pain: A clinical practice guideline from the American College of Physicians	2017	Journal Article	The American College of Physicians	United States	The American College of Physicians	Comprehensive	Low back pain
15. The National Institute for Health and Clinical Excellence	Osteoarthritis in over 16s: Diagnosis and management	2022	Report	The National Institute for Health and Clinical Excellence	United Kingdom	The UK Department of Health and Social Care	Comprehensive	Osteoarthritis
16. Blanpied	Neck pain: Revision 2017 clinical practice guidelines	2017	Report	The American Physical Therapy Association	United States	The American Physical Therapy Association	Comprehensive	Neck Pain
17. The Royal Australian College of General Practitioners	Guideline for the management of knee and hip osteoarthritis	2018	Report	The Royal Australian College of General Practitioners	Australia	The Royal Australian College of General Practitioners	Comprehensive	Hip osteoarthritis; knee osteoarthritis

*CAM* Complementary & alternative medicine, *CPG* Clinical practice guideline, *DoD* Department of Defense, *VA* Veterans Affairs

### Details of acupuncture interventions

Table 2 and Table S1, Supplementary File, present the details of the acupuncture interventions examined in the included CPGs. A total of 35 clinical recommendations were identified across 17 CPGs. Of these, 14 (40.0%) on shoulder pain, 11 (31.4%) focused on low back pain, eight (22.9%) on osteoarthritis, and two (5.7%) on neck pain. Manual or traditional acupuncture was the most frequently discussed intervention type, featured in 26 (74.3%) recommendations, followed by electro-acupuncture (*n* = 10; 28.6%), laser acupuncture (*n* = 4; 11.4%), dry needling (*n* = 4; 11.4%), warm needle acupuncture (*n* = 3; 8.6%), and fire needle (*n* = 2; 5.7%). Nine (25.7%) recommendations involved performing manual acupuncture on specific acupoints (e.g., Ashi points) or with specific needle manipulation techniques (e.g., deep needle insertion). Five (14.3%) recommendations combined acupuncture with physical therapy, self-exercise, or other modalities such as therapeutic ultrasound.

**Table 2:** Details of the interventions examined in the clinical practice guidelines

Variable	*N* (%)
Chronic musculoskeletal pain	
Shoulder pain	14 (40.0)
Low back pain	11 (31.4)
Knee osteoarthritis	5 (14.3)
Hip osteoarthritis	2 (5.7)
Osteoarthritis (not specified)	1 (2.9)
Neck pain	2 (5.7)
Type of acupuncture	
Manual or traditional acupuncture	26 (74.3)
Electro-acupuncture	10 (28.6)
Laser acupuncture	4 (11.4)
Dry needling	4 (11.4)
Warm needle	3 (8.6)
Fire needle	2 (5.7)
Specified acupoint selection	
Yes	9 (25.7)
No	26 (74.3)
Provided instructions on acupuncture administration	
Yes	7 (20.0)
No	28 (80.0)
Provided information on adverse events or harms	
Yes	14 (40.0)
No	21 (60.0)
Provided information on applicable patient population	
Yes	35 (100)
No	0 (0)
Professionals for administering acupuncture	
All clinicians	12 (34.3)
Traditional & complementary medicine practitioners only	11 (31.4)
Medical doctors or specialists only	2 (5.7)
Not specified or not applicable	10 (28.6)
Settings for administering acupuncture	
Traditional & complementary medicine settings	11 (31.4)
Any settings or all levels of care	9 (25.7)
Primary care settings	3 (8.6)
Not specified	12 (34.3)

Twenty-three (65.7%) recommendations lacked details on acupoint selection or implementation specifics, such as patient positioning or intervention duration. In addition, 21 (60.0%) recommendations did not provide information on adverse events or potential harms associated with the interventions. While all clinical recommendations identified the populations for whom the interventions were intended, fewer specified the professionals responsible for administering the interventions (*n* = 25; 71.4%) or the settings in which they should be performed (*n* = 23; 65.7%).

### Details of clinical recommendations

#### Comparators of recommendations and clinical effectiveness of interventions

Table 3 and Table S2, Supplementary File, provide details of the 35 clinical recommendations discussed in the included CPGs. Thirty-eight (80.0%) explicitly mentioned the comparators used in their supporting evidence, with sham acupuncture being the most reported. Other comparators included alternative types of acupuncture that were not the primary interventions or sham interventions, usual care or no treatment, anti-inflammatory or analgesic agents, and physical therapy or orthopaedic treatments. Acupuncture demonstrated significant benefits for pain relief in osteoarthritis, low back pain, neck pain, and shoulder pain across 16 (45.8%) recommendations, with varying effect sizes and durations. Similarly, they showed significant effects on functional improvement for all four conditions in 16 (45.8%) recommendations, while superior effects on quality of life improvement were observed only for low back pain and osteoarthritis in three (8.6%) recommendations.

**Table 3:** Details of the 35 clinical recommendations discussed in the clinical practice guidelines

Variable	*N* (%)
Comparators	
Sham acupuncture	12 (34.3)
Other types of (sham) acupuncture	11 (31.5)
Usual care or no treatment	10 (28.6)
Anti-inflammatory or analgesic agents/injections	3 (8.6)
Exercise, physical therapy, or orthopaedic treatments	3 (8.6)
Not specified	7 (20.0)
Significantly positive clinical effectiveness	
Pain relief	16 (45.8)
Functional improvement	16 (45.8)
Quality of life improvement	3 (8.6)
Assessment system	
GRADE approach	18 (51.5)
Good Practice Point grading system	11 (31.5)
North American Spine Society Levels of Evidence	4 (11.5)
American College of Physicians Grading System	1 (2.9)
Oxford CEBM Levels of Evidence	1 (2.9)
Reported direction of the recommendation	
Recommended or suggested for	21 (60.0)
Recommended or suggested against	6 (17.2)
Did not make recommendations	8 (22.9)
Reported quality of evidence^*^	
High	4 (11.5)
Moderate	8 (22.9)
Low	10 (28.6)
Very low	10 (28.6)
Not reported or not applicable	3 (8.6)
Standardised levels of evidence^†^	
Level 1	3 (8.6)
Level 1-	16 (45.8)
Level 2	3 (8.6)
Level 2-	2 (5.8)
Level 3	0 (0)
Level 4	0 (0)
Level 5	11 (31.5)
Standardised grades of recommendations^†^	
Grade A	3 (8.6)
Grade B	3 (8.6)
Grade C	5 (14.3)
Grade D	24 (68.6)

*CEBM* Centre for Evidence-Based Medicine, *GRADE *Grading of Recommendations Assessment, Development, and Evaluation

^*^When a recommendation was supported by multiple pieces of evidence of varying quality, the lowest quality level was used

^†^Oxford CEBM Levels of Evidence framework was adopted to standardise the levels of evidence and grades of recommendations of clinical recommendations

### Reported direction and strength of recommendations

More than half (*n* = 18; 51.5%) of the clinical recommendations were formulated using the GRADE (Grading of Recommendations Assessment, Development, and Evaluation) approach [Table 3 and Table S2, Supplementary File]. Eleven (31.5%) recommendations from a single CPG were generated using the “Good Practice Point” 5-point grading system developed by its authors. Three other CPGs, respectively, utilised the North American Spine Society Levels of Evidence, the American College of Physicians Grading System, and the Oxford CEBM Levels of Evidence for formulating their recommendations.

Among the 35 clinical recommendations, 21 (60.0%) advised or suggested the use or consideration of acupuncture for managing shoulder pain (*n* = 13; 37.1%), low back pain (*n* = 4; 11.4%), neck pain (*n* = 2; 5.7%), and knee osteoarthritis (*n* = 2; 5.7%). However, only two of the 21 recommendations were strong recommendations, focused on shoulder pain. Eight (22.9%) clinical recommendations did not make explicit suggestions, while six (17.1%) advised against the use of acupuncture. The negative recommendations conflicted with the positive ones, opposing the use of acupuncture for low back pain (*n* = 2; 5.7%), neck pain (*n* = 1; 2.9%), general osteoarthritis (*n* = 1; 2.9%), knee osteoarthritis (*n* = 1; 2.9%), and hip osteoarthritis (*n* = 1; 2.9%). Regarding the reported quality of evidence, only four (11.5%) recommendations were based exclusively on the highest quality evidence, whereas 20 (57.2%) were supported entirely or partially by low- or very low-quality evidence.

### Standardised levels of evidence and grades of recommendations

In terms of the level of evidence for the clinical recommendations, evaluated using the Oxford CEBM approach, only three (8.6%) were classified at Level 1, the highest level [Table 3 and Table S2, Supplementary File]. Eighteen (51.6%) recommendations were rated as Level 1- or Level 2- due to the heterogeneity of the supporting systematic reviews or the heterogeneity of the supporting trials with a high risk of bias, respectively. A total of 11 (31.5%) recommendations were rated at Level 5, primarily due to the inclusion of poor-quality observational studies or reliance solely on clinical opinion. Regarding the grade of recommendation, three (8.6%) clinical recommendations were classified as Grade “A” based on their Level 1 evidence. In contrast, the majority (*n* = 24; 68.6%) were rated Grade “D” due to heterogeneity or Level 5 evidence.

Two of the Grade “A” recommendations were moderate positive suggestions for the use of laser acupuncture and manual acupuncture or electro-acupuncture for chronic low back pain. The remainder made neither positive or negative suggestions on the use of fire needle, electro-acupuncture, or warm needle for hip osteoarthritis.

### Methodological quality of the included clinical practice guidelines

Table 4 summarises the results of the methodological quality appraisal for the 17 included CPGs. The CPGs generally demonstrated high methodological quality, with median scores of ≥ 80.0% across five of the six AGREE II domains. The exception was the “applicability” domain, which had a median score of 58.3%, indicating moderate quality. Within this domain, only four (23.5%) CPGs were rated as high quality, six (35.3%) as moderate quality, and the remainder as low quality. In contrast, a significant proportion of CPGs achieved high-quality ratings in the domains of “scope and purpose” (*n* = 14; 82.4%), “stakeholder involvement” (*n* = 11; 64.7%), “rigour of development” (*n* = 15; 88.2%), “clarity of presentation” (*n* = 14; 82.4%), and “editorial independence” (*n* = 11; 64.7%), with the remaining CPGs in these domains rated as moderate quality. In terms of overall quality, 10 (25.0%) of the included CPGs were rated as high quality, while the remainder were rated as moderate quality, with a median score of 87.5%. A total of six (35.3%) CPGs were recommended for use in their current form by both reviewers, while eight (47.1%) and three (17.6%) were deemed to require modifications before use by one and both reviewers, respectively.

**Table 4:** Results of the methodological quality appraisal for the clinical practice guidelines

Title	Year	Domain 1	Domain 2	Domain 3	Domain 4	Domain 5	Domain 6	Overall quality	Recommendation of use
Traditional Chinese medicine for frozen shoulder: An evidence-based guideline	2023	100.0%	66.7%	92.7%	100.0%	58.3%	75.0%	91.7%	Yes = 0; Yes with modifications = 2
American Academy of Orthopaedic Surgeons management of osteoarthritis of the knee (non-arthroplasty) evidence-based clinical practice guideline	2021	100.0%	66.7%	94.8%	100.0%	81.3%	100.0%	83.3%	Yes = 1; Yes with modifications = 1
Formulation of Japanese Orthopaedic Association (JOA) clinical practice guideline for the management of low back pain - The revised 2019 edition	2022	100.0%	72.2%	66.7%	80.6%	41.7%	87.5%	75.0%	Yes = 1; Yes with modifications = 1
Evidence-based clinical guidelines for multidisciplinary spine care: Diagnosis & treatment of low back pain	2020	100.0%	100.0%	95.8%	55.6%	66.7%	75.0%	91.7%	Yes = 1; Yes with modifications = 1
Non-surgical interventions for lumbar spinal stenosis leading to neurogenic claudication: A clinical practice guideline	2021	100.0%	100.0%	100.0%	94.4%	100.0%	100.0%	100.0%	Yes = 2; Yes with modifications = 0
VA/DoD clinical practice guideline: Diagnosis and treatment of low back pain	2022	100.0%	100.0%	88.5%	100.0%	87.5%	87.5%	91.7%	Yes = 2; Yes with modifications = 0
National clinical guidelines for non-surgical treatment of patients with recent onset neck pain or cervical radiculopathy	2017	83.3%	83.3%	88.5%	97.2%	37.5%	100.0%	75.0%	Yes = 1; Yes with modifications = 1
National clinical guidelines for non-surgical treatment of patients with recent onset low back pain or lumbar radiculopathy	2017	94.4%	94.4%	96.9%	94.4%	33.3%	100.0%	75.0%	Yes = 1; Yes with modifications = 1
Evidence Based (GRADE Approach) Korean Medicine clinical practice guidelines of manual acupuncture for the treatment of shoulder pain	2017	100.0%	94.4%	97.9%	77.8%	31.3%	75.0%	91.7%	Yes = 1; Yes with modifications = 1
VA/DoD clinical practice guideline for the non-surgical management of hip & knee osteoarthritis	2020	83.3%	100.0%	87.5%	94.4%	83.3%	75.0%	91.7%	Yes = 2; Yes with modifications = 0
Low back pain and sciatica in over 16s: Assessment and management	2020	100.0%	100.0%	100.0%	100.0%	100.0%	100.0%	100.0%	Yes = 2; Yes with modifications = 0
Acupuncture for treatment of knee osteoarthritis: A clinical practice guideline	2023	88.9%	88.9%	83.3%	94.4%	58.3%	100.0%	75.0%	Yes = 1; Yes with modifications = 1
PEER simplified chronic pain guideline: Management of chronic low back, osteoarthritic, and neuropathic pain in primary care	2022	75.0%	63.9%	76.0%	100.0%	41.7%	75.0%	58.3%	Yes = 0; Yes with modifications = 2
Noninvasive treatments for acute, subacute, and chronic low back pain: A clinical practice guideline from the American College of Physicians	2017	69.4%	72.2%	87.5%	94.4%	43.8%	75.0%	66.7%	Yes = 0; Yes with modifications = 2
Osteoarthritis in over 16s: Diagnosis and management	2022	100.0%	66.7%	84.4%	66.7%	35.4%	87.5%	75.0%	Yes = 1; Yes with modifications = 1
Neck pain: Revision 2017 clinical practice guidelines	2017	77.8%	80.6%	91.7%	100.0%	62.5%	100.0%	83.3%	Yes = 2; Yes with modifications = 0
Guideline for the management of knee and hip osteoarthritis	2018	100.0%	94.4%	85.4%	100.0%	68.8%	100.0%	91.7%	Yes = 2; Yes with modifications = 0

*DoD *Department of Defense, *VA* Veterans Affairs

## Discussion

### Summary of findings

This study reviewed 17 CPGs with 35 clinical recommendations regarding the use of acupuncture for chronic musculoskeletal pain, published between 2014 and 2024. Among these recommendations, the most frequently addressed condition was shoulder pain and frozen shoulder (40.0%), followed by low back pain and related conditions (31.4%), general, hip, or knee osteoarthritis (22.9%), and neck pain and related conditions (5.7%). Various types of acupuncture were considered, but manual acupuncture was the most discussed, featuring in 26 (74.3%) recommendations. 60% of the clinical recommendations suggested the use of acupuncture, while 22.9% did not make explicit suggestions, and 17.1% advised against its use. The grades of recommendations varied, with only 8.6% achieving the highest grade, while the majority (68.6%) received the lowest grade, indicating the lack of methodological rigour of their supporting evidence. According to the AGREE II appraisal, the 17 CPGs demonstrated high methodological quality, with median scores of ≥ 80.0% across five of the six domains, except for the “applicability” domain.

### Strengths and limitations

This systematic review synthesised acupuncture CPGs for chronic musculoskeletal pain published over the past decade (2014–2024), identified through nine international electronic databases without language restrictions. It extracted details of their clinical recommendations, provided a standardised summary of the levels of evidence supporting these recommendations and their corresponding grades using the Oxford CEBM Levels of Evidence approach, and critically appraised the methodological quality of the CPGs using the AGREE II instrument.

This work has some limitations. First, information in the included publications was insufficient to allow a robust analysis of the factors influencing the direction of recommendations. Second, although we did not impose language restrictions in our inclusion criteria, our search was limited to major English and Chinese databases. This decision assumed that international publications from other countries, such as Japan and Korea, would include English abstracts indexed in these databases. However, this approach may have led to the omission of relevant guidelines published exclusively in other languages without English abstracts. While our search did identify and screen records in Japanese and Korean, none ultimately met our pre-defined eligibility criteria for inclusion. Third, while we have aimed to provide a standardised level for each piece of evidence and a consistent grade for each recommendation to support comparison across the included CPGs, we acknowledge that these may not fully capture the nuances of each guideline. We therefore encourage readers to also consider the strength of recommendations and quality of evidence as reported by the original developers when making decisions.

### Implications

Acupuncture has been recommended for pain management by international and local authorities. However, our study revealed significant discrepancies and contradictions among existing acupuncture CPGs regarding the recommendations for the use of acupuncture in chronic musculoskeletal pain management. One reason for this may be that, in addition to clinical evidence on benefits and harms, guideline developers might also need to consider other factors such as the interventions’ acceptability, feasibility, costs, and the preferences of patients and practitioners. For instance, in regions where CAM is an integral part of mainstream medical practice (e.g., South Korea, the People’s Republic of China), guideline developers might be more inclined to consider acupuncture as a widely accepted and preferred intervention for chronic musculoskeletal pain. Considering the existing infrastructure—both in terms of clinical expertise and physical resources—acupuncture may be deemed feasible for all stakeholders within these communities. Additionally, implementation costs may be relatively low due to its extensive utilisation in these regions. These factors might have played an essential role in recommendation formulation, especially given the lack of high-quality evidence on the effectiveness and safety of acupuncture interventions, as shown in this review and in a recent cross-sectional methodological survey on acupuncture meta-analyses [20]. Therefore, before applying the clinical recommendations to practice, practitioners should evaluate the rationales behind such recommendations and ensure their applicability to their clinical and, more importantly, cultural circumstances [21]. Future studies should also assess the cost-effectiveness of these recommendations to support their implementation in real-world practice. Where appropriate, locally developed or adapted CPGs by committees consisting of local practitioners, field experts, policymakers, patients, and caregivers can better address context-specific factors such as regulatory environments, professional training, patient expectations, and healthcare infrastructure, These committees may utilise the GRADE-ADOLOPMENT framework [22, 23], which offers guidance on adopting existing recommendations from other CPGs, adapting them to developers’ own context, or creating new recommendations de novo.

Another noteworthy observation from this review is that a significant number (65.7%) of clinical recommendations for acupuncture did not provide practical details regarding the specific acupoints for the conditions, the required manipulation techniques and patient positioning, the duration of each treatment session, and the minimum number of sessions needed before reviewing the treatment plan. More importantly, the required frequencies and intensities for electro-acupuncture were not explicitly covered by almost all the recommendations. Without these details, the CPGs may provide little, if any, clinical guidance to practitioners and may, in turn, jeopardise their confidence in resorting to guidelines in general. Future research should focus on the implementation or practical aspect of acupuncture, in addition to its effectiveness and safety, to provide empirical evidence to support the development of CPGs that are more useful for routine practice.

This review synthesised all existing high-quality CPGs on acupuncture for chronic musculoskeletal pain, restricting the scope to those developed by guideline development committees employing robust methodology, with the aim of maximising clinical relevance. Yet, these criteria likely excluded a substantial number of TCM-specific CPGs, which are often formulated through expert consensus without adhering to or reporting the formal methodologies typically used in modern evidence-based guideline development [24]. This raises an important question for researchers and practitioners: how should the balance between expert consensus and clinical evidence be weighted in TCM (and other forms of CAM), and more critically, how can the underlying ideological tensions between traditional and modern medical research be reconciled? Given the increasing familiarity of TCM practitioners with TCM-specific CPGs [24], particularly in light of the recent surge in relevant publications [25], addressing these considerations will be fundamental to advancing evidence-based practice in this field.

Moreover, to support the evaluation of the methodological quality of acupuncture CPGs, we propose the future development of an AGREE II extension tailored to TCM domain [26]. A key addition to the current instrument could be the availability and clarity of reporting on the diagnostic criteria for pattern (or syndrome) differentiation. As a fundamental aspect of TCM and certain other forms of CAM, pattern differentiation involves a comprehensive analysis of a patient’s clinical features to identify the location, cause, and nature of the disease (collectively as the “diagnostic pattern”) [8], aiming to guide the selection of appropriate treatment principles. The extension should also facilitate the critical appraisal of the associations between diagnostic patterns and corresponding treatment principles [26], as well as the location of each acupoint if the Standard Acupuncture Nomenclature is not adopted by the acupuncture CPGs [27]. 

To enhance the adoption of acupuncture CPGs for managing chronic musculoskeletal pain, future research may benefit from incorporating stakeholder interviews guided by established implementation science frameworks, such as the Theoretical Domains Framework (TDF) [28]. The TDF includes 14 domains derived from 128 constructs across 33 theories in health and social psychology related to behaviour change. These domains provide a comprehensive lens through which to examine the behavioural determinants affecting CPG uptake at individual, organisational, and system levels. Building on this, the Behaviour Change Wheel can be applied to conduct a behavioural diagnosis, identifying specific targets for intervention to support the effective integration of CPGs into clinical practice [29]. 

## Conclusions

Significant contradictions exist among recent CPGs on acupuncture for chronic musculoskeletal pain, particularly regarding whether it should be recommended for routine practice, even for the same conditions. Considering the heterogeneity of clinical and cultural contexts, it may be beneficial to develop local CPGs using rigorous methodology, ensuring the involvement of local stakeholders and considering factors such as acceptability, feasibility, costs, and the preferences of both patients and practitioners. A more appropriate tool should also be developed for the rigorous assessment of the methodological quality of acupuncture CPGs.

## Supplementary Information

Supplementary Material 1.

## Data Availability

All data supporting the findings of this study are available within the paper and its Supplementary File.
