# Supplementary material for: Systematic review of clinical practice guidelines on acupuncture for chronic musculoskeletal pain
Source: BMC Complement Med Ther. 2025 Sep 1;25:322. doi: 10.1186/s12906-025-05070-y (PMC12403485; doi:10.1186/s12906-025-05070-y)
Supplement: Supplementary file 1 — Supplementary Material 1. [file 12906_2025_5070_MOESM1_ESM.docx]

**Table S1.** Detailed characteristics of the 17 included clinical practice guidelines

| **Title** | **Chronic musculoskeletal pain** | **Type of acupuncture** | **Acupoints selection** | **Instructions on acupuncture administration** | **Adverse events or harms (related to acupuncture)** | **Applicable population** | **Applicable healthcare professionals** | **Settings (where to use acupuncture)** |
| --- | --- | --- | --- | --- | --- | --- | --- | --- |
| Traditional Chinese medicine for frozen shoulder: An evidence-based guideline | Frozen shoulder | Manual acupuncture | The most used acupoints are LI-15, TB-14, LI-14, SI-9, LU-5, LI-11, TE-5, LI-4, and Ashi point (the point where the participant feels the most pain). | The depth and manipulation of acupuncture depend on the patient’s physique for safety. After eliciting a deqi sensation, the appropriate retention time in acupuncture is generally 20–30 min (once every other day for 3 weeks). | Not reported | People with confirmed frozen shoulder or high-risk of having frozen shoulder | Physicians and related scientific research workers engaged in the clinical work of TCM orthopaedics, integrating traditional Chinese and Western medicine orthopaedics, acupuncture and massage, TCM, rehabilitation, and other clinical work in medical institutions at all levels | Any settings or all levels of care |
| Traditional Chinese medicine for frozen shoulder: An evidence-based guideline | Frozen shoulder | Manual acupuncture combined with modalities (e.g., therapeutic ultrasound, deep heating modalities, shortwave diathermy, electrical stimulation) | The most used acupoints are LI-15, TB-14, LI-14, SI-9, LU-5, LI-11, TE-5, LI-4, and Ashi point (the point where the participant feels the most pain). | The depth and manipulation of acupuncture depend on the patient’s physique for safety. After eliciting a deqi sensation, the appropriate retention time in acupuncture is generally 20–30 min (once every other day for 3 weeks). | Not reported | People with confirmed frozen shoulder or high-risk of having frozen shoulder | Physicians and related scientific research workers engaged in the clinical work of TCM orthopaedics, integrating traditional Chinese and Western medicine orthopaedics, acupuncture and massage, TCM, rehabilitation, and other clinical work in medical institutions at all levels | Any settings or all levels of care |
| Traditional Chinese medicine for frozen shoulder: An evidence-based guideline | Frozen shoulder | Warm needle acupuncture | The most used acupoints are LI-15, TB-14, LI-14, SI-9, LU-5, LI-11, TE-5, LI-4, and Ashi point (the point where the participant feels the most pain). | The depth and manipulation of acupuncture depend on the patient’s physique for safety. After eliciting a deqi sensation, the appropriate retention time in acupuncture is generally 20–30 min (once every other day for 3 weeks). | Not reported | People with confirmed frozen shoulder or high-risk of having frozen shoulder | Physicians and related scientific research workers engaged in the clinical work of TCM orthopaedics, integrating traditional Chinese and Western medicine orthopaedics, acupuncture and massage, TCM, rehabilitation, and other clinical work in medical institutions at all levels | Any settings or all levels of care |
| American Academy of Orthopaedic Surgeons management of osteoarthritis of the knee (non-arthroplasty) evidence-based clinical practice guideline | Knee osteoarthritis | Dry needling | Not specified | Not specified | Not reported | Adults (ages 17 years and older) who have been diagnosed by a trained healthcare provider with osteoarthritis of the knee and are undergoing treatment | Orthopaedic surgeons and other healthcare providers managing patients with osteoarthritis of the knee | Not specified |
| American Academy of Orthopaedic Surgeons management of osteoarthritis of the knee (non-arthroplasty) evidence-based clinical practice guideline | Knee osteoarthritis | Manual acupuncture or electro-acupuncture | Not specified | Not specified | Common side effects can include soreness and minor bleeding or bruising where needles are inserted. | Adults (ages 17 years and older) who have been diagnosed by a trained healthcare provider with osteoarthritis of the knee and are undergoing treatment | Orthopaedic surgeons and other healthcare providers managing patients with osteoarthritis of the knee | Not specified |
| Formulation of Japanese Orthopaedic Association (JOA) clinical practice guideline for the management of low back pain - The revised 2019 edition | Low back pain | Manual acupuncture or electro-acupuncture | Not specified | Not specified | Not reported | People with acute, subacute, and chronic low back pain | Japanese board-certified orthopaedic surgeons and other clinicians, such as primary care physicians | Any settings or all levels of care |
| Evidence-based clinical guidelines for multidisciplinary spine care: Diagnosis & treatment of low back pain | Low back pain | Dry needling | Not specified | Not specified | Not reported | Adults (18 years or older) with low back pain defined as pain of musculoskeletal origin extending from the lowest rib to the gluteal fold that may at times extend as somatic referred pain into the thigh | Not specified | Not specified |
| Evidence-based clinical guidelines for multidisciplinary spine care: Diagnosis & treatment of low back pain | Low back pain | Laser acupuncture (with exercise) | Not specified | Not specified | Not reported | Adults (18 years or older) with low back pain defined as pain of musculoskeletal origin extending from the lowest rib to the gluteal fold that may at times extend as somatic referred pain into the thigh | Not specified | Not specified |
| Evidence-based clinical guidelines for multidisciplinary spine care: Diagnosis & treatment of low back pain | Low back pain | Laser acupuncture (with exercise) | Not specified | Not specified | Not reported | Adults (18 years or older) with low back pain defined as pain of musculoskeletal origin extending from the lowest rib to the gluteal fold that may at times extend as somatic referred pain into the thigh | Not specified | Not specified |
| Evidence-based clinical guidelines for multidisciplinary spine care: Diagnosis & treatment of low back pain | Low back pain | Manual acupuncture or electro-acupuncture | Not specified | Not specified | Not reported | Adults (18 years or older) with low back pain defined as pain of musculoskeletal origin extending from the lowest rib to the gluteal fold that may at times extend as somatic referred pain into the thigh | Not specified | Not specified |
| Non-surgical interventions for lumbar spinal stenosis leading to neurogenic claudication: A clinical practice guideline | Lumbar spinal stenosis | Manual acupuncture | Needle acupuncture (e.g., Hwato Acupuncture, Suzhou, China; 0.30×40 mm/0.30×75 mm) at various sites (e.g., Acupoints of BL-23, BL-25, BL-40, BL-57, and KI-3 or outward from the spinous process bilaterally at L2, L4, S2, and S4, middle of the popliteal fossa, inferior recess in the fibular head, lower end of the groove of the inner and outer head of the gastrocnemius). | Not specified | Minor and transitory undesirable effects (worsening of symptoms, general discomfort, pain at the treated areas, and body ache). | Adults (≥18 years of age) with lumbar spinal stenosis (acquired, congenital, lateral or central) leading to neurogenic claudication with or without associated spondylolisthesis | Rehabilitation clinicians caring for patients with LSS causing NC (e.g., physicians, physiotherapists, chiropractors, occupational therapists, acupuncturists, athletic therapists, massage therapists, nurse practitioners); medical specialists (physiatrists, rheumatologists orthopaedic surgeons, neurosurgeons) | Any settings or all levels of care |
| VA/DoD clinical practice guideline: Diagnosis and treatment of low back pain | Low back pain | Manual acupuncture | Not specified | Not specified | Mostly mild and transient adverse events reported, similar between acupuncture and sham groups. | Adults (aged 18 years or older) with chronic low back pain with or without neurological symptoms, who are eligible for care in the VA or DoD healthcare delivery systems and those who receive care from community-based clinicians. It includes Veterans and Service Members as well as their dependents | VA and DoD primary care providers and others involved in the healthcare team caring for patients with LBP and associated conditions; community-based clinicians involved in the care of Service Members, beneficiaries, or Veterans with low back pain | Any settings or all levels of care |
| National clinical guidelines for non-surgical treatment of patients with recent onset neck pain or cervical radiculopathy | Cervical radiculopathy | Manual acupuncture | Not specified | Not specified | Risk of complications (not specified) | Patients above the age of 18 years symptoms and clinical signs of cervical radiculopathy | Not applicable (recommended against) | Not specified |
| National clinical guidelines for non-surgical treatment of patients with recent onset low back pain or lumbar radiculopathy | Lumbar radiculopathy | Manual acupuncture | Not specified | Not specified | Not reported | Patients with symptoms and clinical signs of lumbar radiculopathy above the age of 18 years. | Not specified | Not specified |
| Evidence Based (GRADE Approach) Korean Medicine clinical practice guidelines of manual acupuncture for the treatment of shoulder pain | Shoulder pain | Manual acupuncture | Not specified | Not specified | Minor adverse events (not specified) | Adults with acute, subacute, or chronic shoulder pain | Traditional Korean medicine doctors | Traditional Korean medicine hospitals and clinics |
| Evidence Based (GRADE Approach) Korean Medicine clinical practice guidelines of manual acupuncture for the treatment of shoulder pain | Shoulder pain | Manual acupuncture (different comparator) | Not specified | Not specified | Minor adverse events (not specified) | Adults with acute, subacute, or chronic shoulder pain | Traditional Korean medicine doctors | Traditional Korean medicine hospitals and clinics |
| Evidence Based (GRADE Approach) Korean Medicine clinical practice guidelines of manual acupuncture for the treatment of shoulder pain | Shoulder pain | Manual acupuncture + physical therapy | Not specified | Not specified | Minor adverse events (not specified) | Adults with acute, subacute, or chronic shoulder pain | Traditional Korean medicine doctors | Traditional Korean medicine hospitals and clinics |
| Evidence Based (GRADE Approach) Korean Medicine clinical practice guidelines of manual acupuncture for the treatment of shoulder pain | Shoulder pain | Manual acupuncture + self-exercise | Not specified | Not specified | Not reported | Adults with acute, subacute, or chronic shoulder pain | Traditional Korean medicine doctors | Traditional Korean medicine hospitals and clinics |
| Evidence Based (GRADE Approach) Korean Medicine clinical practice guidelines of manual acupuncture for the treatment of shoulder pain | Shoulder pain | Manual acupuncture on Ashi points | Ashi points on the coracoid process, humerus lesser tuberosity fracture or humerus greater tuberosity fracture, below the acromion or around the intertubercular groove, or on the maximum tender points in a specific motion or posture causing pain | Not specified | Not reported | Adults with acute, subacute, or chronic shoulder pain | Traditional Korean medicine doctors | Traditional Korean medicine hospitals and clinics |
| Evidence Based (GRADE Approach) Korean Medicine clinical practice guidelines of manual acupuncture for the treatment of shoulder pain | Shoulder pain | Manual acupuncture on both proximal points and distal points | Proximal acupoints: LI-15, TE-14, SI-9, GB-21, SI-14, TE-15, SI-13, SI-11, LI-14, LI-16, and LU-1  Distal acupoints: LI-11, TE-5, LI-4, and SI-3 | Not specified | Not reported | Adults with acute, subacute, or chronic shoulder pain | Traditional Korean medicine doctors | Traditional Korean medicine hospitals and clinics |
| Evidence Based (GRADE Approach) Korean Medicine clinical practice guidelines of manual acupuncture for the treatment of shoulder pain | Shoulder pain | Manual acupuncture with acupoints in the lower limb | Lower limb acupoints: ST38–BL-57, GB-34, SP-9, and SP-6 | Not specified | Not reported | Adults with acute, subacute, or chronic shoulder pain | Traditional Korean medicine doctors | Traditional Korean medicine hospitals and clinics |
| Evidence Based (GRADE Approach) Korean Medicine clinical practice guidelines of manual acupuncture for the treatment of shoulder pain | Shoulder pain | Manual acupuncture with deep needle insertion | Not specified | Deep insertion up to 40 mm deep for myofascial pain and 40–75 mm deep for ST-38–BL-57 acupuncture | Not reported | Adults with acute, subacute, or chronic shoulder pain | Traditional Korean medicine doctors | Traditional Korean medicine hospitals and clinics |
| Evidence Based (GRADE Approach) Korean Medicine clinical practice guidelines of manual acupuncture for the treatment of shoulder pain | Shoulder pain | Manual acupuncture with high-intensity manual stimulation | Not specified | Rotating the needle for 270 degree or more and lifting and thrusting of 10–12 mm or more to induce deqi sensation | Adverse effects reported (not specified) | Adults with acute, subacute, or chronic shoulder pain | Traditional Korean medicine doctors | Traditional Korean medicine hospitals and clinics |
| Evidence Based (GRADE Approach) Korean Medicine clinical practice guidelines of manual acupuncture for the treatment of shoulder pain | Shoulder pain | Manual acupuncture with the Cervical Hyeopcheok (Jiaji) point | EX-B2 | Not specified | Not reported | Adults with acute, subacute, or chronic shoulder pain | Traditional Korean medicine doctors | Traditional Korean medicine hospitals and clinics |
| Evidence Based (GRADE Approach) Korean Medicine clinical practice guidelines of manual acupuncture for the treatment of shoulder pain | Shoulder pain | Manual acupuncture with the Dong's acupuncture method | Not specified | Performing distal acupuncture on the Dong's acupoints on the lower or upper (affected or unaffected) limb before proximal acupuncture | Adverse effects reported (not specified) | Adults with acute, subacute, or chronic shoulder pain | Traditional Korean medicine doctors | Traditional Korean medicine hospitals and clinics |
| VA/DoD clinical practice guideline for the non-surgical management of hip & knee osteoarthritis | Hip osteoarthritis | Fire needle; electro-acupuncture; warm needle acupuncture | Not specified | Not specified | Minor possible side effects (not specified) | Adults aged 18 years or older with osteoarthritis of the hip | All VA and DoD healthcare practitioners involved in the care of adult patients with hip osteoarthritis | Any settings or all levels of care |
| VA/DoD clinical practice guideline for the non-surgical management of hip & knee osteoarthritis | Knee osteoarthritis | Fire needle; electro-acupuncture; warm needle acupuncture | Not specified | Not specified | Minor possible side effects (not specified) | Adults aged 18 years or older with osteoarthritis of the knee | All VA and DoD healthcare practitioners involved in the care of adult patients with knee osteoarthritis | Any settings or all levels of care |
| Low back pain and sciatica in over 16s: Assessment and management | Low back pain | Manual acupuncture | Not specified | Not specified | Not reported | People aged 16 years or above with non-specific low back pain | Not specified | Not specified |
| Acupuncture for treatment of knee osteoarthritis: A clinical practice guideline | Knee osteoarthritis | Manual acupuncture or electro-acupuncture | Local acupoints: Around the diseased knee, including SP-10, EX-LE-4, BL-40, GB-34, SP-9, ST-34, ST-36, and Ashi point  Distal acupoints: Selected according to Jing-jin pattern differentiation and Zang-fu pattern differentiation | Patient position: Determined by the location of selected acupoints and acupuncture manipulation, based on the principle of patient comfort and acupuncturist convenience  Deqi sensation: Both manual acupuncture and electro-acupuncture are requested Deqi, which includes feelings of soreness, numbness, heaviness, and distension  Manipulation: The basic manipulation includes lifting, thrusting, swirling, and rotating  Intensity of electroacupuncture: The muscle around the acupoint has a slight contraction and patient can withstand it  Time of a session: 20–30 min  Frequency: 1–5 sessions per week  Duration: 4–8 weeks | Mild adverse events including dizziness, pain at the needling site, and hematoma | Adult patients with knee osteoarthritis and their families | Physicians, acupuncturists, rheumatologists, orthopaedic surgeons, and rehabilitation physicians | Not specified |
| PEER simplified chronic pain guideline: Management of chronic low back, osteoarthritic, and neuropathic pain in primary care | Low back pain | Electro-acupuncture | Not specified | Not specified | Not reported | Patients with chronic low back pain | Not specified | Not specified |
| Noninvasive treatments for acute, subacute, and chronic low back pain: A clinical practice guideline from the American College of Physicians | Low back pain | Manual acupuncture | Not specified | Not specified | No reported harms or serious adverse events | Adults with chronic (>12 weeks) low back pain | All clinicians | Primary care settings |
| Osteoarthritis in over 16s: Diagnosis and management | Osteoarthritis | Manual acupuncture, electro-acupuncture, or dry needling | Not specified | Not specified | Bruising, bleeding, brief pain worsening after stimulation, and fatigue | Adults (age ≥16 years) with osteoarthritis affecting any joint | Not specified | Not specified |
| Neck pain: Revision 2017 clinical practice guidelines | Neck pain | Dry needling | Not specified | Not specified | Not reported | Adult patients with non-cancer, chronic neck pain with mobility deficits | Not specified | Any settings or all levels of care |
| Guideline for the management of knee and hip osteoarthritis | Knee osteoarthritis | Manual acupuncture, electro-acupuncture, or laser acupuncture | Not specified | Not specified | Not reported | Adults diagnosed with symptomatic osteoarthritis of the knee up until referral for joint replacement | General practitioners | Primary care settings |
| Guideline for the management of knee and hip osteoarthritis | Hip osteoarthritis | Manual acupuncture, electro-acupuncture, or laser acupuncture | Not specified | Not specified | Not reported | Adults diagnosed with symptomatic osteoarthritis of the hip up until referral for joint replacement | General practitioners | Primary care settings |

DoD: Department of Defense; TCM: Traditional Chinese medicine; VA: Veterans Affairs

**Table S2.** Details of the 35 clinical recommendations discussed in the clinical practice guidelines

| **Title** | **Chronic musculoskeletal pain** | **Type of acupuncture** | **Comparator** | **Outcome & Clinical effectiveness** | **Adopted assessment system** | **Reported recommendation & strength** | **Reported quality of evidence** | **Other factors considered** | **Levels of Evidence^*^** | **Grades of Recommendation^*^** |
| --- | --- | --- | --- | --- | --- | --- | --- | --- | --- | --- |
| Traditional Chinese medicine for frozen shoulder: An evidence-based guideline | Frozen shoulder | Manual acupuncture | Not specified | Pain and function (flexion, extension, abduction, internal rotation, and external rotation) (significant benefits) | GRADE approach | We suggest using acupuncture to decrease pain VAS scores and improve the range of flexion, extension, abduction, internal rotation, and external rotation for frozen shoulder patients at any clinical stage. (Weak or conditional recommendation) | Low to very low quality of evidence | Certainty of evidence; costs; clinical feasibility; accessibility; clinical acceptability | 1- (SR of RCTs with high heterogeneity) | D |
| Traditional Chinese medicine for frozen shoulder: An evidence-based guideline | Frozen shoulder | Manual acupuncture combined with modalities (e.g., therapeutic ultrasound, deep heating modalities, shortwave diathermy, electrical stimulation) | Not specified | Pain and function (significant benefits) | GRADE approach | We suggest integrating acupuncture and modalities to decrease pain VAS scores and improve Melle scores for frozen shoulder patients at any clinical stage. (Weak or conditional recommendation) | Low to very low quality of evidence | Certainty of evidence; costs; clinical feasibility; accessibility; clinical acceptability | 1- (SR of RCTs with high heterogeneity) | D |
| Traditional Chinese medicine for frozen shoulder: An evidence-based guideline | Frozen shoulder | Warm acupuncture | Not specified | Pain and function (significant benefits) | GRADE approach | We suggest using warm acupuncture to decrease pain VAS scores and improve shoulder function rating scale scores in each dimension for frozen shoulder patients at any clinical stage. (Weak or conditional recommendation) | Low to very low quality of evidence | Certainty of evidence; costs; clinical feasibility; accessibility; clinical acceptability | 1- (SR of RCTs with high heterogeneity) | D |
| American Academy of Orthopaedic Surgeons management of osteoarthritis of the knee (non-arthroplasty) evidence-based clinical practice guideline | Knee osteoarthritis | Manual acupuncture or electro-acupuncture | No acupuncture; sham acupuncture; sham TENS; usual care | Pain and function (inconsistent evidence) | GRADE approach | Acupuncture may improve pain and function in patients with knee osteoarthritis. (Limited recommendation) | High to moderate quality of evidence | Acceptability; feasibility | 1- (SRs of RCTs with high heterogeneity) | D |
| American Academy of Orthopaedic Surgeons management of osteoarthritis of the knee (non-arthroplasty) evidence-based clinical practice guideline | Knee osteoarthritis | Dry needling | Exercise; sham dry needling or manual therapy | Pain and function (inconsistent evidence) | GRADE approach | In the absence of reliable evidence, it is the opinion of the workgroup that the utility/efficacy of dry needling is unclear and requires additional evidence. (Consensus recommendation) | High quality of evidence | Not reported | 5 (Recommendation based on clincical opinion) | D |
| Formulation of Japanese Orthopaedic Association (JOA) clinical practice guideline for the management of low back pain - The revised 2019 edition | Low back pain | Manual acupuncture or electro-acupuncture | Not specified | Function and quality of life (significant effects in the short term) | GRADE approach | There is no established evidence of complementary and alternative medicine therapies (including acupuncture) in Japan. Therefore, it is basically impossible to state their usefulness or recommendation. | Not reported | Not reported | 1- (SR of RCTs with high heterogeneity) | D |
| Evidence-based clinical guidelines for multidisciplinary spine care: Diagnosis & treatment of low back pain | Low back pain | Laser acupuncture | Sham acupuncture | Pain and function (little to no effect) | North American Spine Society Levels of Evidence | Laser acupuncture provides no short-term or medium-term benefit over sham treatment for patients with chronic low back pain. (Recommended) | Good quality of evidence | Not reported | 1 | A |
| Evidence-based clinical guidelines for multidisciplinary spine care: Diagnosis & treatment of low back pain | Low back pain | Manual acupuncture or electro-acupuncture | Usual care | Pain and function (significant benefits in the short term but no effect in the long term) | North American Spine Society Levels of Evidence | In patients with chronic low back pain, addition of acupuncture to usual care is recommended for short-term improvement of pain and function compared to usual care alone. (Insufficient evidence) | Good quality of evidence | Not reported | 1 | A |
| Evidence-based clinical guidelines for multidisciplinary spine care: Diagnosis & treatment of low back pain | Low back pain | Dry needling | Not specified | Pain and function (conflicting results) | North American Spine Society Levels of Evidence | There is insufficient evidence for or against the use of dry needling as a treatment option for patients with chronic low back pain. (Insufficient evidence) | Good to fair quality of evidence | Not reported | 2 (RCT with high risk of bias) | B |
| Evidence-based clinical guidelines for multidisciplinary spine care: Diagnosis & treatment of low back pain | Low back pain | Manual acupuncture or electro-acupuncture | Sham acupuncture | Pain and function (conflicting results) | North American Spine Society Levels of Evidence | In patients with low back pain, there is conflicting evidence that acupuncture provides improvements in pain and function as compared to sham acupuncture. (Recommended) | Good to fair quality of evidence | Not reported | 2- (RCTs with high risk of bias and high between-study heterogeneity) | D |
| Non-surgical interventions for lumbar spinal stenosis leading to neurogenic claudication: A clinical practice guideline | Lumbar spinal stenosis | Manual acupuncture | Sham acupuncture; usual care | Pain and function (marginal effects in the short term but no effects in the long term) | GRADE approach | We suggest considering traditional acupuncture on a trial basis. (Conditional/Weak) | Very low quality of evidence | Expert opinion; patient values and preferences; resource implications | 2- (RCTs with high risk of bias and high between-study heterogeneity) | D |
| VA/DoD clinical practice guideline: Diagnosis and treatment of low back pain | Low back pain | Manual acupuncture | Sham acupuncture | Pain (small benefit in the intermediate term but no significant difference in the long term)  Function (conflicting results) | GRADE approach | For patients with chronic low back pain, we suggest acupuncture. (Weak for) | Low quality of evidence | Patient or provider values and preferences; resource use; equity; acceptability; feasibility; subgroup considerations | 1- (SR of RCTs with high heterogeneity) | D |
| National clinical guidelines for non-surgical treatment of patients with recent onset neck pain or cervical radiculopathy | Cervical radiculopathy | Manual acupuncture | Usual care | No valid/relevant supporting evidence | GRADE approach | It is not good clinical practice to routinely offer acupuncture in patients with recent onset cervical radiculopathy in addition to other treatment. (Consensus recommendation) | Quality of evidence rating not applicable | Quality of evidence; patient values and preferences; perception and experience of the working groups | 5 (Recommendation based on clinical opinion) | D |
| National clinical guidelines for non-surgical treatment of patients with recent onset low back pain or lumbar radiculopathy | Lumbar radiculopathy | Manual acupuncture | Usual care | No valid/relevant supporting evidence | GRADE approach | It is not good practice to offer acupuncture on a routine basis to patients with recent onset lumbar nerve root compression. (Consensus recommendation) | Quality of evidence rating not applicable | Quality of evidence; patient values and preferences; perception and experience of the working groups | 5 (Recommendation based on clinical opinion) | D |
| Evidence Based (GRADE Approach) Korean Medicine clinical practice guidelines of manual acupuncture for the treatment of shoulder pain | Shoulder pain | Manual acupuncture | Sham acupuncture | Pain and function (little to no effect) | 5-point grading system; Good Practice Point | Strongly recommended. | Moderate quality of evidence | Patient or provider values and preferences; cost of treatment; acceptability; feasibility | 1- (SR of RCTs with high heterogeneity) | D |
| Evidence Based (GRADE Approach) Korean Medicine clinical practice guidelines of manual acupuncture for the treatment of shoulder pain | Shoulder pain | Manual acupuncture + self-exercise | Self-exercise + ultrasonic treatment; steroid injection | Function (no significant effect until 12 months when compared to self-exercise and ultrasonic treatment but had significant effects at 12 months)  Pain and function (higher treatment effects when compared to steroid injection) | 5-point grading system; Good Practice Point | Weakly recommended. | Moderate quality of evidence | Patient or provider values and preferences; cost of treatment; acceptability; feasibility | 1- (SR of RCTs with high heterogeneity) | D |
| Evidence Based (GRADE Approach) Korean Medicine clinical practice guidelines of manual acupuncture for the treatment of shoulder pain | Shoulder pain | Manual acupuncture + physical therapy | TENS + physical therapy | Pain and function (significant effects until 6 months) | 5-point grading system; Good Practice Point | Strongly recommended. | High quality of evidence | Patient or provider values and preferences; cost of treatment; acceptability; feasibility | 2 (RCT with high risk of bias) | B |
| Evidence Based (GRADE Approach) Korean Medicine clinical practice guidelines of manual acupuncture for the treatment of shoulder pain | Shoulder pain | Manual acupuncture | Conservative orthopaedic treatment (not specified) | Pain and function (conflicting results) | 5-point grading system; Good Practice Point | Unable to offer a recommendation. | Moderate quality of evidence | Patient or provider values and preferences; cost of treatment; acceptability; feasibility | 2 (RCT with high risk of bias) | B |
| Evidence Based (GRADE Approach) Korean Medicine clinical practice guidelines of manual acupuncture for the treatment of shoulder pain | Shoulder pain | Manual acupuncture with high-intensity manual stimulation | Manual acupuncture with low-intensity manual stimulation | No valid/relevant supporting evidence | 5-point grading system; Good Practice Point | May be considered. | Low quality of evidence | Patient or provider values and preferences; cost of treatment; acceptability; feasibility | 5 (Non-RCTs with poor quality) | C |
| Evidence Based (GRADE Approach) Korean Medicine clinical practice guidelines of manual acupuncture for the treatment of shoulder pain | Shoulder pain | Manual acupuncture with Ashi points | Manual acupuncture without Ashi points | No valid/relevant supporting evidence | 5-point grading system; Good Practice Point | May be considered. | Low quality of evidence | Patient or provider values and preferences; cost of treatment; acceptability; feasibility | 5 (Non-RCTs with poor quality) | C |
| Evidence Based (GRADE Approach) Korean Medicine clinical practice guidelines of manual acupuncture for the treatment of shoulder pain | Shoulder pain | Manual acupuncture with the Dong's acupuncture method | Manual acupuncture without the Dong's acupuncture method | No valid/relevant supporting evidence | 5-point grading system; Good Practice Point | May be considered. | Low quality of evidence | Patient or provider values and preferences; cost of treatment; acceptability; feasibility | 5 (Non-RCTs with poor quality) | C |
| Evidence Based (GRADE Approach) Korean Medicine clinical practice guidelines of manual acupuncture for the treatment of shoulder pain | Shoulder pain | Manual acupuncture with deep needle insertion | Manual acupuncture with shallow needle insertion | Pain and function (conflicting results) | 5-point grading system; Good Practice Point | Weakly recommended. | Moderate quality of evidence | Patient or provider values and preferences; cost of treatment; acceptability; feasibility | 5 (Non-RCTs with poor quality) | C |
| Evidence Based (GRADE Approach) Korean Medicine clinical practice guidelines of manual acupuncture for the treatment of shoulder pain | Shoulder pain | Manual acupuncture on both proximal points and distal points | Manual acupuncture on proximal points only | No valid/relevant supporting evidence | 5-point grading system; Good Practice Point | Weakly recommended. | Low quality of evidence | Patient or provider values and preferences; cost of treatment; acceptability; feasibility | 5 (Recommendation based on clinical opinion) | D |
| Evidence Based (GRADE Approach) Korean Medicine clinical practice guidelines of manual acupuncture for the treatment of shoulder pain | Shoulder pain | Manual acupuncture with acupoints in the lower limb | Manual acupuncture without acupoints in the lower limb | No valid/relevant supporting evidence | 5-point grading system; Good Practice Point | Weakly recommended. | Low quality of evidence | Patient or provider values and preferences; cost of treatment; acceptability; feasibility | 5 (Recommendation based on clinical opinion) | D |
| Evidence Based (GRADE Approach) Korean Medicine clinical practice guidelines of manual acupuncture for the treatment of shoulder pain | Shoulder pain | Manual acupuncture with the Cervical Hyeopcheok (Jiaji) point | Manual acupuncture without the Cervical Hyeopcheok (Jiaji) point | No valid/relevant supporting evidence | 5-point grading system; Good Practice Point | May be considered. | Low quality of evidence | Patient or provider values and preferences; cost of treatment; acceptability; feasibility | 5 (Recommendation based on clinical opinion) | D |
| VA/DoD clinical practice guideline for the non-surgical management of hip & knee osteoarthritis | Hip osteoarthritis | Fire needle; electro-acupuncture; warm needle | Not specified | Pain and function (acupuncture had little to no effect when compared to sham acupuncture and other acupuncture interventions) | GRADE approach | There is insufficient evidence to recommend for or against the use of complementary and integrative health interventions (including acupuncture) for the treatment of osteoarthritis of the hip. (Neither for nor against) | Very low quality of evidence | Patient or provider values and preferences; resource use; equity; acceptability; feasibility; subgroup considerations | 1 | A |
| VA/DoD clinical practice guideline for the non-surgical management of hip & knee osteoarthritis | Knee osteoarthritis | Fire needle; electro-acupuncture; warm needle | Not specified | Pain and function (fire needle and electro-acupuncture had statistically significant improvements when compared to other acupuncture interventions)  Pain and function (warm needle had no significant improvements when compared to other acupuncture interventions) | GRADE approach | There is insufficient evidence to recommend for or against the use of complementary and integrative health interventions (including acupuncture) for the treatment of osteoarthritis of the knee. (Neither for nor against) | Very low quality of evidence | Patient or provider values and preferences; resource use; equity; acceptability; feasibility; subgroup considerations | 1- (SR of RCTs with high heterogeneity) | D |
| Low back pain and sciatica in over 16s: Assessment and management | Low back pain | Manual acupuncture or electro-acupuncture | Sham acupuncture; usual care; waiting list control | Pain and function among population without sciatica (no significant difference when compared to sham acupuncture)  Quality of life - Composite physical score among population without sciatica (clinically important benefit when compared to sham acupuncture)  Quality of life - Composite mental score among population without sciatica (no significant difference when compared to sham acupuncture)  Pain and function among population with or without sciatica (no significant difference when compared to sham acupuncture)  Pain among population without sciatica (clinically important benefit when compared to usual care up to 4 months)  Function among population without sciatica (clinically important benefit when compared to usual care in the short term)  Quality of life - Composite physical score among population without sciatica (clinically important benefit when compared to usual care up to 4 months)  Pain and function among population with or without sciatica (clinically important benefit when compared to usual care in the short term but not the long term)  Quality of life - All domains among population with or without sciatica (clinically important benefit when compared to usual care in the short term but not the long term)  Quality of life - Composite physical score among population with or without sciatica (clinically important benefit when compared to waiting list control in the short term but not the long term)  Quality of life - Composite mental score among population with or without sciatica (no significant difference when compared to waiting list control)  Pain and function (unclear benefit when compared to TENS and NSAIDs) | GRADE approach | Do not offer acupuncture for managing low back pain with or without sciatica. (Strength of recommendation not specified) | High to very low quality of evidence (manual acupuncture or electro-acupuncture compared to sham acupuncture among population with sciatica)  Moderate to low quality of evidence (manual acupuncture or electro-acupuncture compared to sham acupuncture among population with or without sciatica)  High to very low quality of evidence (manual acupuncture or electro-acupuncture compared to usual care among population with sciatica)  Moderate to very low quality of evidence (manual acupuncture or electro-acupuncture compared to usual care among population with or without sciatica)  Moderate to low quality of evidence (manual acupuncture or electro-acupuncture compared to waiting list control among population with or without sciatica)  Low quality of evidence (manual acupuncture or electro-acupuncture compared to TENS and NSAIDs) | Cost of treatment; psychological distress; healthcare utilisation | 1- (SRs of RCTs with high heterogeneity) | D |
| Acupuncture for treatment of knee osteoarthritis: A clinical practice guideline | Knee osteoarthritis | Manual acupuncture or electro-acupuncture | No treatment; usual care; TENS; NSAIDs | Pain and function (beneficial effect when compared to no treatment)  Function (beneficial effect when compared to usual care)  Pain (no significant effect when compared to usual care) | GRADE approach | We suggest acupuncture rather than no treatment in knee osteoarthritis among adults. (Weak recommendation) | Moderate to low quality of evidence | Availability; cost of treatment; patient values and preferences; clinical feasibility | 1- (SR of RCTs with high heterogeneity) | D |
| PEER simplified chronic pain guideline: Management of chronic low back, osteoarthritic, and neuropathic pain in primary care | Low back pain | Electro-acupuncture | Sham acupuncture | Pain (no significant effect) | GRADE approach | We suggest that electro-acupuncture (unclear benefit) could be discussed with patients when interventions with clear evidence of benefit have already been considered. (Strength of recommendation not specified) | Very low quality of evidence | Data quality; cost of treatment; patient preferences and values; equity; feasibility; acceptability | 1- (SR of RCTs with high heterogeneity) | D |
| Noninvasive treatments for acute, subacute, and chronic low back pain: A clinical practice guideline from the American College of Physicians | Low back pain | Manual acupuncture | No acupuncture; sham acupuncture | Pain and function (moderate effect when compared to no acupuncture)  Pain (moderate effect when compared to sham acupuncture)  Function (no clear effect when compared to sham acupuncture) | American College of Physicians Grading System | For patients with chronic low back pain, clinicians and patients should initially select nonpharmacologic treatment with exercise, multidisciplinary rehabilitation, acupuncture, mindfulness-based stress reduction, tai chi, yoga, motor control exercise, progressive relaxation, electromyography biofeedback, low-level laser therapy, operant therapy, cognitive behavioural therapy, or spinal manipulation. (Strong recommendation) | Moderate quality of evidence | Not reported | 5 (Non-RCTs with poor quality) | C |
| Osteoarthritis in over 16s: Diagnosis and management | Osteoarthritis | Manual acupuncture, electro-acupuncture, or dry needling | Sham acupuncture; no treatment; comparisons between acupuncture interventions | Pain, function, and quality of life (manual acupuncture had no clinically important difference when compared to sham acupuncture)  Pain, function, and quality of life (manual acupuncture had unclear effect in the short term when compared to no treatment and no clinically important difference in the long term)  Pain, function, and quality of life (electro-acupuncture had no clinically important difference when compared to manual acupuncture)  Pain (electro-acupuncture had clinically important benefit in the short term when compared to sham acupuncture but no clinically important difference in the long term)  Function (electro-acupuncture had unclear effect in the short term when compared to sham acupuncture and no clinically important difference in the long term)  Quality of life (electro-acupuncture had clinically important difference in the short term when compared to sham acupuncture but no clinically important difference in the long term)  Pain and function (electro-acupuncture had clinically important benefit in the short term when compared to no treatment)  Quality of life (electro-acupuncture had unclear effect in the short term when compared to no treatment) | GRADE approach | Do not offer acupuncture or dry needling to manage osteoarthritis. (Strength of recommendation not specified) | Moderate to very low quality of evidence (manual acupuncture compared to no treatment)  High to low quality of evidence (electro-acupuncture compared to acupuncture)  High to very low quality of evidence (electro-acupuncture compared to sham acupuncture)  Moderate to very low quality of evidence (electro-acupuncture compared to no treatment) | Cost of treatment; psychological distress; osteoarthritis flares | 1- (SR of RCTs with high heterogeneity) | D |
| Neck pain: Revision 2017 clinical practice guidelines | Neck pain | Dry needling | Control; non-trigger point dry needling; manual acupuncture; wet needling; miniscalpel needling; lidocaine injection; NSAIDs | Pain (clinical benefit over the immediate and short term when compared to control)  Pain and function (clinical benefit over the short term when compared to non-trigger point dry needling and manual acupuncture)  Pain (no clinical benefit over the immediate or intermediate term when compared to wet needling)  Pain (no clinical benefit over the short term when compared to miniscalpel needling and lidocaine injection)  Quality of life (no clinical benefit over the short term when compared to lidocaine injection and NSAIDs)  Pain (no clinical benefit over the immediate term when compared to lidocaine injection) | Oxford CEBM Levels of Evidence | For patients with chronic neck pain with mobility deficits, clinicians should provide a multimodal approach of (1) thoracic manipulation and cervical manipulation or mobilisation, (2) mixed exercise for cervical/scapulothoracic regions, and (3) dry needling, laser, or intermittent traction. (Moderate recommendation) | Levels of evidence III to IV | Not reported | 1- (SR of RCTs with high heterogeneity) | D |
| Guideline for the management of knee and hip osteoarthritis | Knee osteoarthritis | Manual acupuncture, electro-acupuncture, or laser acupuncture | Sham acupuncture | Pain (manual acupuncture had moderate, statistically significant benefits within 8 to 13 weeks but no significant benefits within 26 weeks)  Function and quality of life (manual acupuncture had no significant benefits within 8 to 13 weeks and within 26 weeks)  Pain (electro-acupuncture had small, statistically significant benefits by 26 weeks)  Function and quality of life (electro-acupuncture had no significant benefits by 26 weeks)  Pain, function, and quality of life (laser acupuncture had no significant benefits) | GRADE approach | We suggest not offering acupuncture (i.e., traditional, laser, electro) for people with knee osteoarthritis. (Conditional against recommendation) | Low quality of evidence | Cost of treatment | 1- (SR of RCTs with high heterogeneity) | D |
| Guideline for the management of knee and hip osteoarthritis | Hip osteoarthritis | Manual acupuncture, electro-acupuncture, or laser acupuncture | Sham acupuncture | Pain and function (manual acupuncture, electro-acupuncture, and laser acupuncture had small statistically significant benefits) | GRADE approach | We suggest not offering acupuncture (i.e., traditional, laser, electro) for people with hip osteoarthritis. (Conditional against recommendation) | Very low quality of evidence | Cost of treatment | 1- (SR of RCTs with high heterogeneity) | D |

CEBM: Centre for Evidence-Based Medicine; DoD: Department of Defense; GRADE: Grading of Recommendations Assessment, Development, and Evaluation; NSAID: Non-steroidal anti-inflammatory drug; RCT: Randomised controlled trial; SR: Systematic review; TENS: Transcutaneous electrical nerve stimulation; VA: Veterans Affairs; VAS: Visual Analogue Scale

^*^Oxford CEBM Levels of Evidence framework was adopted to standardise the levels of evidence and grades of recommendations of clinical recommendations
